# Using health data for decision-making at each level of the health system to achieve universal health coverage in Ethiopia: the case of an immunization programme in a low-resource setting

**DOI:** 10.1186/s12961-021-00694-1

**Published:** 2021-08-11

**Authors:** Binyam Tilahun, Alemayehu Teklu, Arielle Mancuso, Berhanu F. Endehabtu, Kassahun D. Gashu, Zeleke A. Mekonnen

**Affiliations:** 1 Department of Health Informatics, Institute of Public Health, College of Medicine and Health Sciences, University of Gondar, Gondar, Ethiopia; 2 eHealthlab Ethiopia, Institute of Public Health, College of Medicine and Health Sciences, University of Gondar, Gondar, Ethiopia; 3 Department of Pediatrics, School of Medicine, University of Gondar, Gondar, Ethiopia; 4 Alliance for Health Policy and Systems Research, World Health Organization, Geneva, Switzerland; 5 Health System Directorate, Ministry of Health, Addis Ababa, Ethiopia

**Keywords:** Data use, Data quality, Accountability, Implementation research, Ethiopia

## Abstract

**Background:**

For evidence-based decision-making, there is a need for quality, timely, relevant and accessible information at each level of the health system. Limited use of local data at each level of the health system is reported to be a main challenge for evidence-based decision-making in low- and middle-income countries. Although evidence is available on the timeliness and quality of local data, we know little about how it is used for decision-making at different levels of the health system. Therefore, this study aimed to assess the level of data use and its effect on data quality and shared accountability at different levels of the health system.

**Methods:**

An implementation science study was conducted using key informants and document reviews between January and September 2017. A total of 21 key informants were selected from community representatives, data producers, data users and decision-makers from the community to the regional level. Reviewed documents include facility reports, district reports, zonal reports and feedback in supervision from the district. Thematic content analysis was performed for the qualitative data.

**Results:**

Respondents reported that routine data use for routine decision-making was low. All health facilities and health offices have a performance monitoring team, but these were not always functional. Awareness gaps, lack of motivating incentives, irregularity of supportive supervision, lack of community engagement in health report verification as well as poor technical capacity of health professionals were found to be the major barriers to data use. The study also revealed that there are no institutional or national-level regulations or policies on the accountability mechanisms related to health data. The community-level Health Development Army programme was found to be a strong community engagement approach that can be leveraged for data verification at the source of community data.

**Conclusion:**

The culture of using routine data for decision-making at the local level was found to be low. Strengthening the capacity of health workers and performance monitoring teams, introducing incentive mechanisms for data use, engaging the community in data verification and introducing accountability mechanisms for health data are essential to improve data use and quality.

## Background

Primary healthcare services, such as immunizations, are fundamental to improving health and health equity, particularly in the context of low- and middle-income settings where resources are scarce. Health programs in low- and middle-income countries need evidence-based decision-making to effectively deliver health services to all citizens with the available limited resources [1].

Evidence-based decision-making also requires a coordinated effort on the part of data producers and users from multiple levels of the health information system [1]. Ethiopia follows a three-tier health system comprising the primary health care unit as primary level (primary hospital, health center and health post), the general hospital as secondary level and the referral hospital as tertiary level. The reporting system follows a hierarchical approach where health data is reported on a monthly basis. Ethiopia has made substantial progress on universal health coverage although the health information system still needs improvement [2]. As the lower levels of the health system are the primary source of the data, there is a need for strong mechanisms to ensure the availability, quality and use of data. The availability of high-quality health data enables health planners, managers and healthcare providers to make decisions based on evidence at all levels of the health system. At higher levels, aggregated data are needed for strategic policy-making and resource allocation [3].

Evidence-informed decision-making relies on the availability of high-quality evidence. Conversely, the lack of routine data use at all levels is a main system failure leading to the poor availability and poor quality of data in low- and middle-income countries. Evidence shows that low-resource settings often have limited use of local data for health system planning and decision-making [4–6]. In Ethiopia, the prevailing practices in terms of the effective utilization of data for decision-making are not satisfactory, and the quality of the health data is an unsolved problem [2, 4]. Improving the relationship of data quality, demand for data and data use creates a cycle that potentially leads to improved health programmes and policies [7].

The weak use of data can be attributed to a high degree of fragmentation across multiple parallel information subsystems, lack of community engagement and severely constrained information system infrastructure and human resources [2]. An assessment of data quality and information use conducted in 2012 in Ethiopia also showed a limited culture of data use for decision-making, with only 37% of the facilities implementing discussion and decisions based on findings from routine health information [4]. Another study of the health information system in Ethiopia showed data management and use for decision-making to be inadequate at lower levels [6, 8].

Immunization coverage has improved worldwide in the past years, however the validity of the data for measuring change over time has been questioned [9]. To meet this problem, donor-supported initiatives have made efforts to assess the quality of data used [10]. Data from the Ethiopian Demographic and Health Survey (EDHS) reports generally show vaccination coverage to be lower than that reported in the routine service statistics of the Ministry of Health. This raises questions on data quality and the reporting of problems in the health system [11]. A comparative analysis conducted by the United States Agency for International Development (USAID) on immunization data in rural health posts in Ethiopia indicated that there was a 12% disparity in complete vaccination coverage between the routine Health Management Information System (HMIS) and survey coverage, indicating data quality problems. Programme assessments revealed that reports were occasionally fabricated in some facilities because of additional incentive needs as promotion packages. At the district level, the most common challenge was reporting data to the next level without or with minimal value for decision-making in that particular district [11–13].

Evidence also shows that organizational and technical factors can affect the culture of data use and quality [14–16]. The practical utility of health information is determined by multiple factors that can be categorized into three general categories: the attitudes and actions of people who produce or use data, the technical aspects of data processes and tools and the organizational context that supports data processes [5, 12].

Evidence indicates that the quality of data, and consequently the information system, must be seen in a broader perspective that not only focuses on technicalities (data collection tools and the reporting system) but also on support mechanisms [4, 8]. In Ethiopia, different reports show that the quality and use of the data are still not improving as intended [17]. Data quality in the immunization programme in Ethiopia is poor; in part causing limited data use at all levels.

The use of health data for decision-making and actions to improve the quality of health services and to achieve performance goals is also the vital ingredient of accountability in the health sector [18]. However, the main cause for the lack of data use is not yet thoroughly studied. Moreover, little is known about data use at each level of the health system. In this study, we aim to investigate the different factors affecting the use of data at each level of the health system in Ethiopia, which in turn is affecting the performance of the health system and progress towards universal health coverage in the Amhara region, northwest Ethiopia.

## Objectives

The main objective of this study was assess the level of data use at each level of the health system in Ethiopia and its effect on data quality, accountability and overall health system performance.

### Specific objectives

1. To explore how data are reported and used for decision-making at each level of the health system, from the community to the district and national levels.
2. To explore supportive supervision practices, interaction across levels of the health system and feedback mechanisms within the health information system actors at the district, facility and community levels.
3. To explore existing community-level engagement approaches that can be leveraged to increase data use, improve data quality and ensure accountability in the health system.

## Methods

### Study protocol

The study design and the methods were peer-reviewed, and the protocol was published in the* BMC Health Research Policy and Systems* journal [19].

### Study setting

This study was conducted in the Wogera and Dabat districts of North Gondar Zone, north-west Ethiopia. The North Gondar Zone has 22 districts and 557 Kebele (small administrative units).

### Study design and period

An implementation research study was conducted using mixed methods, from January to September 2017. The study aimed to address the underlying implementation barriers and facilitators of immunization health data quality and use. We used key informant interviews to gain an in-depth understanding of data reporting, supervision and feedback mechanisms in the immunization programme, as well as as the underlying factors affecting data use for decision-making. In addition, we undertook a document review of secondary data. A total of 21 key informants were included in the study: seven from the community, six from health centers, six from Woreda, one from the North Gondar Zonal Health Department and one from the Amhara Regional Health Bureau.

### Data collection tools

We used a semi-structured interview guide to assess the organizational, behavioural and technical factors for data use, data quality and related issues based on the Performance of Routine Information System Management (PRISM) framework. Data extraction sheets were used for capturing report completeness and feedbacks provided.

### Qualitative data

Qualitative data was gathered through key informant interviews using a semi-structured guide with open-ended and closed questions. Data producers, data users, community representatives and decision-makers were included in the key informant interview.

The key informant interview tool for decision-makers comprised 25 questions relating to the level of use of information for decision-making. The key informant interview tool for data producers also comprised 25 questions and focused on the flow of information and the interaction between support mechanisms. The key informant interview tool for data users comprised 32 questions relating to the role of data users. Finally, the key informant interview tool for community representatives comprised 33 questions on the level of involvement in health data decision-making.

### Data collection techniques

#### Key informant interviews

A preliminary test was carried out to validate the interview tools. Members of the research team conducted face-to-face interviews with four groups of participants (data producers, data users, community representatives and decision-makers) from the district, facility and community selected using purposive sampling. The interviews involved 21 participants, including district managers, facility-level data users and supervisors, data producers and community representatives. The interviews were carried out by two people in the research team, and sessions were recorded with respondents’ consent. Interviews were conducted at locations and times convenient to key stakeholders, thus ensuring their privacy and confidentiality. Data collections were done using the local language (Amharic) and transcribed for analysis.

#### Document review

The document review was conducted at each level in the study setting. This review considered the 12 selected health facilities and health offices in the study area. Documents included in the review were facility reports, district reports, zonal reports and district feedback reports. A data extraction sheet was used to extract data from the reports.

### Operational definition

Health facilities in this study refers to health centres and hospitals. The District Health Office is an administrative unit that coordinates the functionality of health facilities under its catchment. The Health Development Army (HDA) is a network of unpaid women volunteers organized which falls under the supervision of health extension workers (HEWs), with the aim to promote health and prevent disease through community participation and empowerment. One HDA comprises 30 members, with each member representing five other groups (1–5 networks) from respective villages. The 30 members serve under the direct supervision of a HEW.

The accountability mechanism in this study means that the responsible body at each level of the health system shall abide by a guideline with the legal framework to control data quality and information use.

### Data analysis

In the qualitative data analysis, audio data was transcribed in Amharic and translated into the English language. The interview verbatim and field notes from the document review were compiled and reviewed by three research team members. The qualitative data were subjected to a thematic analysis. Codes were identified and categorized into the chosen themes. A descriptive analysis was conducted to assess report completeness and feedback provision status. In addition, the perception of study participants towards data quality and data use was analysed and reported in the form of percentages.

### Ethical approval on the study

Ethical approval for this research was obtained from the Research Ethics Review Committee of WHO (reference number ERC 0002865). Local ethical approval for this research was also obtained from the Institutional Ethical Review Board of University of Gondar (reference number VP/RCS/05/165). In addition, written informed consent was provided by interview participants

## Results

### Characteristics of study participants

A total of 21 key informants were included in the study, of whom 13 were men and eight were women. Among these key informants, nine had work experience of 3–4 years (Table 1).

**Table 1:** Characteristics of the study participants

Characteristics	Number
Sex	
Male	13
Female	8
Age (years)	
≤ 34	11
35–44	7
≥ 45	3
Responsibilities	
HMIS officer/M&E	6
EPI focal	4
HEW	2
Organization head/vice-head	3
Kebele administrator	2
HDA leader	4
Years in service	
≤ 2	5
3–4	9
≥ 5	7
Administration level	
Kebele	13
District	5
Zone	1
Region	2
Total	21

*EPI* Expanded Programme on Immunization, *HDA* Health Development Army, *HEW* Health extension worker,* HMIS* Health management information system, *M&E* monitoring and evaluation

### Data quality and data use status

Respondents reported that the use of data for decision-making was low at all levels of the health system, especially at the district level. The majority of respondents expressed concerns about the quality of the data being used for decision-making. The study found that performance monitoring teams (PMTs) are in place at all health facilities, both at the Woreda and zonal level, but that they are not routinely functional. All respondents replied that they have data verification mechanisms for the immunization programme through the PMTs.

#### At the data producers and data user levels

Poor data quality and a low data use culture were the major problems reported by all health information technicians (HITs) at health facilities at the district and zonal levels. The major problems mentioned include data non-timeliness, inaccuracy and incompleteness. In terms of data quality, the HITs reported problems with false reporting, mainly in the form of inflated reports, which affect evidence-based decision-making.

One respondent mentioned that: “*Yes, there is fabrication in reports …*”

#### At the decision-maker level

All decision-makers believed that the level of data use is low in the health sector. One of the obstacles to the use of data for decision-making was reported to be a gap in the awareness of health professionals on information use. There is also a capacity gap in the use of available data for decision-making across the health sector. In terms of capacity for data management and use for decision-making, a high turnover of staff is the major challenge that the regional health bureau is facing.

A respondent from the regional health bureau mentioned that: “*For example, last year we trained all focal persons and when we did quick assessment at the end of the year around 62% has left from their permanent working health facilities. When untrained health workers were hired, they face difficulties to manage the data properly.*”

There is also capacity gap to use the available data for decision-making across the health sector.

One respondent said: “*We have skill gaps in using the data. Skill to use computers is also necessary. For example, we have organizational information since 2007 where eHMIS started. If you asked me data before 2007 I can’t give you.*”

The majority of participants also indicated that the system is generally weak in terms of documenting and using the existing health data.

One respondent explained that: “*The culture of information use is very poor at all levels starting from kebele. We are not using data for decision-making. No feedback systems based on evidence generated. We have also skill gaps in using the data. This is mainly associated with negligence, which is again related with incentives. In terms of commitment, the health workers have overload of activities and became negligent which is frustrating.*”

Another respondent also commented that: "*At facility level, though they are expected to use data for local decision it is not done routinely. For example, health facilities did not use EPI monitoring chart evidences for immediate decision-making. In terms of providing feedbacks still there are gaps.*”

In the document review, we observed that there was an overall 0–11% content completeness of data elements and 0–4% representative completeness of the expected reports from health facilities for the immunization programme. This was also revealed at the zonal level, with 11 out of 22 districts reporting Penta-I vaccination coverage above 100% (ranging between 103 and 112%). This was supported by the qualitative findings where a respondent said that:

*“We evaluated poor quality of immunization data (false report) like; rota-2 Vs Penta-3, we expect rota-2 to be greater than penta-3, however, the report showed the reverse. We even observed measles coverage higher than Rota-2 which is unlikely.”*

The major determinants of data quality and data use are described in three themes in the following sections.

### Role of data use for data quality

At the community level, the majority of respondents strongly agreed that using data for decision-making can improve data quality. Likewise, all decision-makers believed that data use has positive role to play in improving data quality.

In line with this, a respondent mentioned that: “*The culture of data use is very poor at all levels. The more we use data we had better know its function. Then we take care of quality. The more we ignore data use, we can’t understand its benefit then we don’t care about its quality.*”

### Role of supervision and feedback mechanisms in data use

#### At the data producers’ and data users’ level

Among the data producers, half of them strongly agreed that supervisors give due attention to the quality of Expanded Programme on Immunization (EPI) data and provide feedback to EPI reports, while half of them somewhat disagreed on this issue. Similarly, half of data users strongly agreed that supervisors give due attention to the quality of EPI data, followed by one third of them who somewhat agreed.

The respondents reported that supportive supervision is not specific to the EPI and supervision is integrated with other programmes. Participants from the visited health facilities also reported that there were no regular supportive supervisions from higher levels, including Woreda, Zone and regional health bureaus.

A respondent indicated: “*We didn’t receive any supportive supervision from my district last year, they only come for reports. But we have got little supportive supervision from zonal and regional health bureaus. They come with checklists and didn’t focus on data quality and use.*”

Feedbacks were routinely given orally, and on rare occasions in written form, in monthly reports and conducted supportive supervisions.

#### At the decision-makers’ level

Supervisors use integrated checklists during supervision, which included data quality as one component. Most decision-makers give regular feedback to their staff. After approval of the final report, health facilities and health offices send reports to all concerned bodies. Respondents’ perception was that sharing health information has no risk since reports are shared with organizations that support the EPI. Respondents also believed that sharing the reports increases transparency.

### Community-level engagement and accountability system

This study revealed that community engagement in local decision-making was limited to annual planning and performance evaluations.

One respondent mentioned that: “*We are invited in annual planning and evaluation meetings and not involved in other activities. If we were involved in verifying reports which are actually done about our community, we could have verified to avoid false reporting.*”

All respondents said that there is no system that makes health workers accountable for their work, and they could not recall any measures taken so far following their poor performance or false reporting of services to higher levels.

#### At the data producers’ and users’ level

At data producer’s level, all respondents reported that the role of data use in ensuring accountability is very high. However, there was no written rule and no regulation to ensure accountability on health data management in all institutions.

One respondent said that: “*If the decision is based on evidence, there will not be problem and it helps to ensure accountability. Ensuring accountability is important. First, we need to work on the health workers on the importance of data/information. Health workers focus on the technical work and ignore the data/information use. Even, the health worker and the health facility head sent inconsistent reports without verifying and correcting it at local level.*”

#### At the decision-makers’ level

Regarding the establishment of an accountability system, the majority reported that there is no accountability system and written document for data quality of health services and particularly for the immunization programme. Hence, no measure has been taken despite feedbacks having been provided for the problems that occurred. Some of the organizations have included data quality as one parameter for performance evaluation.

One health centre head indicated that: “*Documents are signed for each health worker based on the balanced score card system. They have agreements and the first criteria are to provide services with quality information as core component.*”

## Discussion

Access and utilization of quality data can improve the efficiency and quality of healthcare delivery. All respondents agreed that information is crucial for decision-making. They also agreed that using data/information for decision-making can improve data quality at all levels, although current practices do not achieve this. A comparative analysis done by USAID on immunization data in rural health posts showed similar findings [12].

Poor data quality was a problem reported by all HITs at health facilities and at the district and zonal levels. A study conducted by the Ethiopian Public Health Institute also reported similar findings where data quality and utilization of health information remains weak, particularly at primary health care facilities and district levels [13]. The study findings also indicated inaccuracies in current EPI data. In the document reviews at the zonal level, half of the 22 districts reported above 100% coverage of Penta-I vaccination. These inaccuracies could be due to a denominator problem in addition to the reporting problems, or they could be due to underplanning by EPI service users caused by inappropriate use of the conversion factor for the eligible population. One possible solution to improve estimates of the eligible population could be to use local data during the planning process.

The value of health data and information is determined by its utilization in decision-making [16]. In terms of data use for decision-making, respondents agreed that the availability of accurate and timely information plays an important role in decision-making. The majority of the respondents replied that they have data verification mechanisms for the immunization programme through performance monitoring teams but that the quality of data was still low. A study conducted by Ethiopian Public Health Institute also reported similar findings on data verification and revealed that incomplete and poor quality reports compromise decisions and the allocation of the already scarce resources [13].

This study also found that some of the underlying factors contributing to delayed reporting to the district and zonal levels were poor infrastructure, lack of and/or interruptions to electricity and lack of internet connectivity and transportation facilities. Those facilities with relatively poor access to overall infrastructure tend to delay in submitting their reports.

This study points to capacity and technical gaps in the use of available data for decision-making. A high turnover of staff is the major challenge that the Amhara Regional Health Bureau is facing in terms of data management and information use for decision-making. In general, all functions of the health system rely on the availability of timely, accurate and dependable information for decision-making. In line with this, the need for organized, accessible, timely and accurate data for health decision-making is affected by various factors at different levels, including ICT infrastructure, technical skills and behavioural factors [14].

All decision-makers believed that information use has a positive impact to bring on data quality. Data of poor quality will not be used, and because such data are not used, the data will remain of poor quality. Conversely, greater use of data will help to improve the quality of the data, which will in turn lead to more data use [2].

Verification and feedback systems also improve the quality of data and improve the effectiveness of local and hierarchical utilization of information [4]. The present study establishes that there is no supportive supervision specific to the EPI which focused on data quality and information use. Other studies also showed that data quality assurance and feedback mechanisms were weak and that errors in reporting were due to the lack of supervision and feedback from higher administrative levels [6, 20, 21]. Supportive supervision could improve data quality and information use by capacitating health workers in problem-solving skills and their decision-making capacity in their routine activities.

Participants mentioned that there are currently no community data quality monitoring tools or supervisory and feedback mechanisms in place to scrutinize and enhance data quality, specifically for the immunization programme. Respondents from the visited health facilities and health offices routinely send reports to all concerned bodies. Respondents believe that sharing health information has no risk and can even support EPI and improve transparency.

The use of health data for decisions and actions to improve the quality of health services and to achieve performance goals is the vital ingredient of shared accountability [18]. However, most respondents reported that there is no accountability system and written document for data quality of health services, specifically for the immunization programme. When it comes to taking action, the health system is largely accountable to act and make services available and accessible to the community. Accountability of the health system also lies in making sure that the services provided by the health system are of good quality and provided in a compassionate and respectable manner [18]. All participants reported that there is currently no system that makes health workers accountable for their work, and no one could recall a situation in which measures were taken due to poor performance or false reporting. Healthcare data are more than an asset that lies on the heart of accountability. It helps the health system to improve quality of care and evaluate healthcare costs and service performance; however, in reality healthcare data lack accountability and governance [8].

### Limitation of the study

Although the study used mixed methods, there might be recall and social desirability bias.

## Conclusions

The culture of using routine data for decision-making at the local level was reported to be poor in Ethiopia. Routine use of data for decision-making has a positive role in improving the quality of data and shared accountability. Integrated supportive supervision was not regular at all levels. Community engagement was found to be poor, and there is no policy in place to ensure accountability in the health system. Capacity-building of health workers and PMTs, introducing incentives, engaging the community in data verification and introducing accountability mechanisms are essential to improve data use and thus overall health system performance. Developing community data quality monitoring tools and supervisory mechanisms are also important.

## Data Availability

The datasets used during the current study are available from the corresponding author on reasonable request.

## Abbreviations

EPI: Expanded Programme on Immunization
HIT: Health information technician
PMTs: Performance monitoring teams

## References

[1] MEASURE Evaluation. Data demand and information use in the health sector: a conceptual framework. 2006. https://www.measureevaluation.org/resources/publications/ms-06-16a. Accessed 16 Feb 2017.

[2] Ethiopian Federal Ministry of Health. Information revolution roadmap. 2016.

[3] Nutley T, Reynolds HW (2013). Improving the use of health data for health system strengthening. Glob Health Action.

[4] Mesfin G, Tesfaye MH, Worku N, Mamo D, Fatoumata NT. Data quality and information use: a systematic review to improve evidence, Ethiopia. Afr Health Monitor. 2012;14(2012):53–60.

[5] Avan BI, Berhanu D, Umar N, Wickremasinghe D, Schellenberg J (2016). District decision-making for health in low-income settings: a feasibility study of a data-informed platform for health in India, Nigeria and Ethiopia. Health Policy Plan.

[6] Mengiste S (2010). Analysing the challenges of IS implementation in public health institutions of a developing country: the need for flexible strategies. J Health Inform Dev Ctries.

[7] Abouzahr C, Boerma T. Policy and practice health information systems: the foundations of public health. Bull World Health Organ. 2005;83(8):578–83. PMC2626318 16184276

[8] Teklegiorgis K, Tadesse K, Mirutse G, Terefe W (2016). Level of data quality from Health Management Information Systems in a resources limited setting and its associated factors, eastern Ethiopia. SA J Inf Manag.

[9] WHO/UNICEF. Global immunization data 2014. 2015. Geneva: WHO/UNICEF. pp. 2–5. https://www.who.int/immunization/monitoring_surveillance/global_immunization_data.pdf. Accessed 20 Feb 2017.

[10] Federal Ministry of Health. Ethiopia National Expanded Programme on Immunization comprehensive multi-year plan. 2006–2010.

[11] Central Statistical. Agency. Ethiopian Demographic and Health Survey. 2016. https://dhsprogram.com/pubs/pdf/FR328/FR328.pdf.

[12] JSI Research and Training Institute. Extended Program on Immunization (EPI) coverage in selected Ethiopian zones: a baseline survey for L10K’s routine immunization improvement initiative. 2015. https://l10k.jsi.com/Resources/Docs/EPI-baseline-report.pdf. Accessed 21 Feb 2017.

[13] Ethiopian Public Health Institute. Ethiopia health data quality review: system assessment and data verification for selected indicators. 2016. http://repository.iifphc.org/handle/123456789/132. Accessed 25 Feb 2017.

[14] Aqil A, Lippeveld T, Hozumi D (2009). PRISM framework: a paradigm shift for designing, strengthening and evaluating routine health information systems. Health Policy Plan.

[15] Marquez L, Kean L (2002). Making supervision supportive and sustainable: new approaches to old problems. Maximising Access Qual Pap.

[16] Adamki M, Asamoah D, Riverson K. Assessment of data quality on expanded programme on immunization in Ghana: the case of New Juaben Municipality. J Health Med Inform. 2015;6:2.

[17] Braa J, Heywood A, Sahay S (2012). Improving quality and use of data through data-use workshops: Zanzibar, United Republic of Tanzania. Bull World Health Organ.

[18] Brinkerhoff D (2003). Accountability and health systems: overview, framework.

[19] Tilahun B, Teklu A, Mancuso A, Abebaw Z, Dessie K, Zegeye D. How can the use of data within the immunisation programme be increased in order to improve data quality and ensure greater accountability in the health system? A protocol for implementation science study. Health Res Policy Syst. 2018;16(1):37. 10.1186/s12961-018-0312-2 PMC5934902 29724235

[20] Bailey C, Blake C, Schriver M, Cubaka VK, Thomas T, Hilber AM (2016). A systematic review of supportive supervision as a strategy to improve primary healthcare services in sub-Saharan Africa. Int J Gynaecol Obstet.

[21] Mavimbe JC, Braa J, Bjune G (2005). Assessing immunization data quality from routine reports in Mozambique. BMC Public Health.

